# Insulin-like Growth Factor II mRNA-Binding Protein 1 Regulates Pancreatic Cancer Cell Growth through the Surveillance of *CDC25A* mRNA

**DOI:** 10.3390/cancers15204983

**Published:** 2023-10-13

**Authors:** Davide Di Fusco, Maria Teresa Segreto, Giulia Di Maggio, Andrea Iannucci, Claudia Maresca, Antonio Di Grazia, Marco Colella, Carmine Stolfi, Giovanni Monteleone, Ivan Monteleone

**Affiliations:** 1 Department of Systems Medicine, University of “Tor Vergata”, 00133 Rome, Italy; davidedifusco@gmail.com (D.D.F.); mariateresasegreto13@gmail.com (M.T.S.); dimaggiogiulia3@gmail.com (G.D.M.); maresca.9595@gmail.com (C.M.); adigrazia2000@yahoo.it (A.D.G.); marco040590@gmail.com (M.C.); carmine.stolfi@uniroma2.it (C.S.); gi.monteleone@med.uniroma2.it (G.M.); 2 Department of Biomedicine and Prevention, University of “Tor Vergata”, 00133 Rome, Italy; andreaiannucci93@gmail.com

**Keywords:** pancreatic cancer, IMP1, IGF2BP1, cell death, *CDC25A*, CDK2, cell cycle, RBP, PDAC, RNA-binding protein

## Abstract

**Simple Summary:**

The progression of PDAC is also regulated at the transcription/transduction stage. RNA metabolism is manipulated by different RNA-binding proteins (RBPs), and insulin-like growth factor 2 mRNA-binding proteins (IMPs), such as IMP1, have been associated with a poor prognosis in PDAC patients; however, little is known about its contribution to PDAC carcinogenesis. Public data suggest that PDAC patients with higher IMP1 expression showed poor overall survival. IMP1 silencing leads to reduced cell growth in PDAC cells and three-dimensional spheroids. Abrogation of IMP1 in PDAC cells showed lower levels of *CDC25A*, increased phosphorylation of the cyclin-dependent kinase (CDK)2, and accumulation of cells in the G1 phase. Immunoprecipitation revealed that IMP1 binds *CDC25A* mRNA, thereby controlling cell cycle progression. Ultimately, we proved that suppression of IMP1 blocked in vivo growth of Panc-1 transferred into immunodeficient mice. Our results indicate that IMP1 drives the PDCA cell cycle and represents a novel strategy for overcoming PDCA cell proliferation.

**Abstract:**

A number of data indicate that the sources of different kinds of PDAC may be discovered at the transcription/transduction stage. RNA metabolism is manipulated at various steps by different RNA-binding proteins (RBPs), and the deregulation or irregular activity of RBPs is known to contribute to tumor promotion and progression. The insulin-like growth factor 2 mRNA-binding protein family (IMPs), and IMP1 in particular, has been linked with a poor prognosis in PDAC patients; however, little is known about its contribution in PDAC carcinogenesis. In this study, we investigated the function of IMP1 in PDAC. To evaluate IMP1 expression and correlation with PDAC prognosis, we utilized several public databases. Using a specific siRNA IMP1, we analyzed cell death and cell cycle progression in PDAC cell lines and 3D spheroids. The role of IMP1 was also evaluated in vivo in a Panc-1-derived tumor xenograft murine model. Public data suggest that PDAC patients with higher expression of IMP1 showed poor overall and progression-free survival. IMP1 silencing leads to reduced cell growth in PDAC cells and three-dimensional spheroids. Abrogation of IMP1 in PDAC cells showed lower levels of *CDC25A*, increased phosphorylation of the cyclin-dependent kinase (CDK)2, and accumulation of PDAC cells in the G1 phase. Immunoprecipitation experiments revealed that IMP1 binds *CDC25A* mRNA, thus controlling cell-cycle progression. Ultimately, we proved that suppression of IMP1 blocked in vivo growth of Panc-1 transferred into immunodeficient mice. Our results indicate that IMP1 drives the PDCA cell cycle and represents a novel strategy for overcoming PDCA cell proliferation.

## 1. Introduction

Pancreatic ductal adenocarcinoma (PDAC) is the most prevalent pancreatic neoplastic disease, and PDAC is the fourth most common cause of cancer-related deaths worldwide with a 5-year overall survival of less than 8% [1,2]. The incidence of PDAC is expected to increase further in the future, and projections indicate a more than two-fold increase in the number of cases in the next ten years [1,3]. PDAC carcinogenesis begins with somatic mutations that trigger epigenetic modifications, but they are unable to explain the vast heterogeneity observed among PDAC patients. Instead, epigenetic changes drive transcriptomic alterations that can regulate the malignant phenotype, suggesting that the causes of PDAC heterogeneity can be discovered at the transcription level [4]. Indeed, chemo-resistance and poor outcomes are related to significant transcriptome heterogeneity detected in PDAC [5], and three different PDAC subtypes centered on transcriptomic data have been recently identified [6,7,8]. These studies suggest that the carcinogenesis of PDAC is started by mutations causing epigenetic deregulation that leads to transcriptome modifications which finally reveal the PDAC subtype. mRNA transcription/transduction is controlled at different levels, including RNA-dependent management by short- and long-noncoding RNAs and RNA-binding proteins (RBPs). RBPs are present in various tissues and cell populations and have been evolutionarily saved to maintain their function in basic cellular functions [9]. Different disorders, including cancer, could be triggered by a substantial change or alteration in the RBPs that regulate these crucial cellular functions [10,11]. The deregulation or aberrant function of RBPs is known to contribute to carcinogenesis, and several pieces of evidence suggest that patient prognoses correlate with this unusual expression in cancer tissue [12,13]. Some RBPs also play a role in promoting PDAC tumors, including HuR (ELAVL1), APOBEC1, and PTBP3 [14]. The insulin-like growth factor 2 mRNA-binding protein family (IMP), which represents a group of oncofetal RBPs that control the localization, translation, and stability of several mRNAs, has recently been illustrated as being up-regulated and linked to a poor prognosis in PDAC patients [14,15,16]. A recent article systematically explored the altered expression and prognostic significance of 44 RBPs in PDAC using publicly available data, suggesting that the IMP RBP family, IMP1 and IMP3, are oncogenic candidates in PDAC [14]. However, while an important role for IMP3 in the control of PDAC tumor progression by enhancing the pro-metastatic behavior of tumor cells is known [17], little is known about the role of IMP1 in PDAC progression, despite abundant data suggesting its real impact on the prognosis of PDAC patients. Therefore, here we analyzed the role of IMP1 in PDAC carcinogenesis by using human cell lines and a xenograft mouse model.

## 2. Materials and Methods

### 2.1. Cell Culture

All reagents were purchased from Sigma-Aldrich unless otherwise indicated. Human PDAC cell lines Panc-1 and MIA PaCa-2 were obtained from the American Type Culture Collection (ATCC, Manassas, VA, USA) and cultured in Dulbecco’s modified Eagle’s high glucose medium (DMEM, Gibco, Gaithersburg, MD, USA). The medium was supplemented with 10% fetal bovine serum (FBS), 1% penicillin/streptomycin (all from Lonza, Verviers, Belgium). Cells were maintained in a 37 °C, 5% CO_2_, fully humidified incubator.

To evaluate effective down-regulation of IMP1, cells were incubated with a specific IMP1 inhibitor BTYNB (final concentration 10 μM; from MCE, Monmouth Junction, NJ, USA) or transfected with commercial IMP1 siRNA (ranging from 5 nmol/L to 25 nmol/L) or with a IMP1 antisense oligonucleotide (5′-CTCTCGTTGAGGTTGCC-3′, 2 μg/mL; Integrated DNA Technologies, Coralville, IA, USA) or a control siRNA (final concentration: 25 nmol/L) in presence or absence of 5-fluoruracil or oxaliplatin (both at final concentration of 10 μM) using Opti-MEM medium and lipofectamine 3000 reagent (both from Thermo Fisher Scientific, Waltham, MA, USA), according to the manufacturer’s guidelines.

### 2.2. 3D Culture

2000 cells/well of Panc-1 were seeded into ultra-low attachment 96-well round-bottom plates, cultured as indicated previously, and 3D cultures were maintained for 7 days, obtaining spheroids. Then, the medium was replaced with fresh medium, and, to evaluate effective down-regulation of IMP1, cells were transfected with commercial IMP1 siRNAs for 3 days, as indicated previously.

### 2.3. Protein Extraction and Western Blotting

Total proteins were extracted in lysis buffer as previously specified [11]. Nitrocellulose filters were incubated with primary antibodies against human IMP1 (1:500 final dilution, Abcam, Cambridge, UK), (p)-CDK2 (Thr-14/Tyr-15), CDK2, and *CDC25A* (1:500 final dilution, all from Santa Cruz Biotechnology, Santa Cruz, CA, USA), and then incubated with a secondary antibody conjugated to horseradish peroxidase (HRP)-conjugated secondary antibody with a final dilution (Dako, Milan, Italy). Subsequently, each blot was stripped and then incubated with b-actin antibody (1:5000 final dilution) as a control, to establish equivalent loading of the WB lanes. Computer-assisted scanning densitometry was used to evaluate the amount of the immunoreactive bands of all WB panels (Total Lab, AB.EL Science-Ware Srl, Rome, Italy).

### 2.4. Flow Cytometry Analysis

Cells were left untreated or treated with either control siRNA (final concentration 25 nmol/L) or IMP1 siRNA (final concentration 25 nmol/L) with or without Z-VAD-FMK (final concentration: 20 μmol; R&D Systems, Minneapolis, MN, USA). Cells were collected, washed twice in Annexin V (AnnV) buffer, stained with FITC-AnnV (final dilution: 1:100; Immunotools, Friesoyte, Germany) according to the manufacturer’s guidelines, and incubated with propidium iodide (PI) (5 mg/mL) for 30 min at 4 °C. Cell death was quantified using flow cytometry. For cell-cycle distribution, cells were untreated or transfected with CTR siRNA or IMP1 siRNA (final concentration 25 nmol/L) for 48 h and analyzed as previously indicated [11]. Cells were analyzed using flow cytometry Gallios and Kaluza software Version 2.1 (Beckman Coulter Life Sciences, Pasadena, CA, USA).

### 2.5. RNA Immunoprecipitation

RNA immunoprecipitation of RNA was evaluated in Panc-1 cells using a Magna RIP RNA-binding protein immunoprecipitation kit, according to the manufacturer’s guidelines (Merck KGaA, Darmstadt, Germany). Beads were conjugated with a specific anti-IMP1 antibody (Abcam) or control isotype. The RNA co-immunoprecipitated was isolated and analyzed using RT-PCR. Sequences of the primers used were the following: human *CDC25A* forward 5′-TCCGAGTCAACAGATTCAGGT-3′; reverse 5′-GAAGCCATCATCCTCATCAGA-3′; *TLR2* forward 5′-TGTGCTGTGCTCTGTTTCCTG-3′; reverse 5′-TTCCTGGGCTTCCTTTTGGC-3′; *GAPDH* forward 5′-TGACGTGCCGCCTGGAGAAA-3′; and reverse 5′-AGTGTAGCCCAAGATGCCCTTCAG-3′.

### 2.6. RNA Extraction, Real-Time qPCR

Total RNA was extracted using the PureLink RNA kit (Thermo Fisher Scientific), and reverse transcription and RT-PCR were performed as previously indicated [11]. RNA expression was deduced to the housekeeping *β-actin* signal (forward 5′-AAGATGACCCAGATCATGTTTGAGACC-3′ and reverse 5′-AGCCAGTCCAGACGCAGGAT-3′) on the basis of the Delta-DeltaCT. Human *CDC25A* primers used were as indicated before.

### 2.7. In Vivo Formation of Panc-1-Derived Tumors

In vivo formation of Panc-1 cell-derived tumors was assessed as previously indicated [12]. Panc-1 (5 × 10^5^) was injected subcutaneously, and after one week mice with comparable tumor sizes were divided into two groups, receiving daily intraperitoneal injection (IP) of control sense (S) or IMP1 antisense oligonucleotide (AS), (both at 100 μg/mouse resuspended in 300 mL of PBS; 5′-CTCTCGTTGAGGTTGCC-3′; Integrated DNA Technologies, Coralville, Iowa). To evaluate the efficient delivery of AS in the tumor mass in preliminary experiments, mice were IP injected with fluorescein isothiocyanate (FITC)-conjugated IMP1 AS (100 μg/mouse) or PBS as control; 24 h later, FITC-IMP1 AS distribution in the tumor mass was measured using immunofluorescence. All mice were killed at day 21, and the tumors were isolated and measured as previously indicated [12], and used for protein expression or histological analyses. All in vivo experiments were carried out in strict accordance with Italian and European legislation on animal experimentation (Number of protocol 494/2017-PR).

### 2.8. Statistical Analysis

Data in the present study are displayed as mean ± standard deviation (SD) or ± standard error of the mean (SEM). Student-t and Mann–Whitney tests were used for statistical examination; *p*-values were considered statistically significant with *p* < 0.05.

### 2.9. Data Availability

Public repository data are available at Tumour Immune Estimation Resource (TIMER 2.0) http://timer.cistrome.org/ (accessed on 10 August 2023), the Gene Expression Profiling Interactive Analysis (GEPIA) http://gepia.cancer-pku.cn/index.html (accessed on 10 August 2023), (UALCAN) http://ualcan.path.uab.edu/ (accessed on 10 August 2023), (Human Protein Atlas database) https://www.proteinatlas.org/ (accessed on 10 August 2023) and Post-Transcriptional Regulation (POSTAR3) http://111.198.139.65/index.html (accessed on 10 August 2023).

## 3. Results

### 3.1. IMP1 Is Up-Regulated in Human PDAC and Is Associated with a Poor Prognosis in PDAC Patients

**Figure 1:**
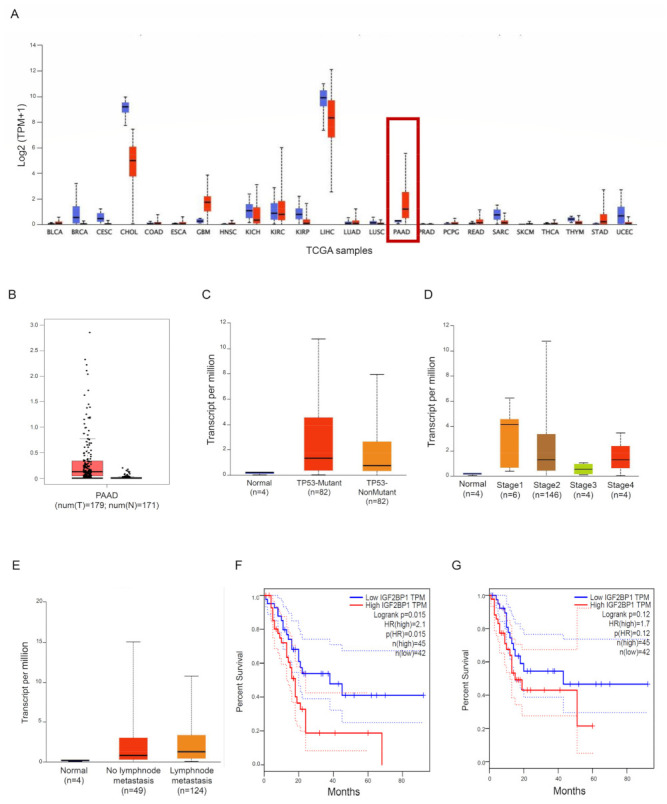
IMP1 expression is increased in PDAC tissue. (**A**) IMP1 expression in several types of human cancer was explored with the TIMER database; red box: normal pancreatic tissue vs. PDAC tissue. (**B**) Expression of IMP1 mRNA in PDAC tissue and normal tissues using the GEPIA database. Box plots indicate IMP1 expression among distinct groups of patients based on clinical parameters: TP53 mutation (**C**), cancer stage (**D**), and nodal metastasis status (**E**) using the UALCAN online tool. Kaplan–Meier plotters are displayed for overall survival (**F**) and disease-free (**G**) survival using the GEPIA database.

We used some publicly accessible datasets to explore the expression of IMP1 in PDCA tissue. Two different public databases, the Tumour Immune Estimation Resource (TIMER; http://timer.cistrome.org/ [18], accessed on 30 August 2023) and the Gene Expression Profiling Interactive Analysis database (GEPIA; http://gepia.cancer-pku.cn/detail.php?gene=IGF2BP1###, accessed on 30 August 2023) [19], showed that IMP1 mRNA was strongly expressed in some solid cancers, including PDAC, matching with normal tissues (Figure 1A,B). We then used the UALCAN online dataset to evaluate IMP1 expression among groups of patients corresponding to different clinical parameters (http://ualcan.path.uab.edu/, accessed on 5 September 2023 [20]). IMP1 expression does not correlate with TP53-mutant or TP53 wild-type PDAC patients (Figure 1C, https://ualcan.path.uab.edu/cgi-bin/TCGAExResultNew2.pl?genenam=IGF2BP1&ctype=PAAD, accessed on 5 September 2023), and a significant increase in IMP1 expression was observed in PDAC patients in tumor stages 1, 2, and 4 (Figure 1D, https://ualcan.path.uab.edu/cgi-bin/TCGAExResultNew2.pl?genenam=IGF2BP1&ctype=PAAD, accessed on 5 September 2023). IMP1 expression was higher in patients with no regional lymph node metastasis and with metastases in axillary lymph nodes compared to normal controls (Figure 1E, https://ualcan.path.uab.edu/cgi-bin/TCGAExResultNew2.pl?genenam=IGF2BP1&ctype=PAAD, accessed on 5 September 2023). These findings suggested that there is a close correlation between IMP1 expression and cancer development from the first stages to metastasis, so we investigated the prognostic value in patients affected by PDAC using the GEPIA database. PDAC patients who express high levels of IMP1 showed poor overall survival (OS, http://gepia.cancer-pku.cn/detail.php?gene=CDC25A###, accessed on 5 September 2023) and progression-free survival (PFS, http://gepia.cancer-pku.cn/detail.php?gene=CDC25A###, accessed on 5 September 2023) (Figure 1F,G), indicating that increased IMP1 in cancer tissue correlates with a poor prognosis in PDAC patients.

### 3.2. IMP1 Sustains PDAC Cell Survival and Proliferation

**Figure 2:**
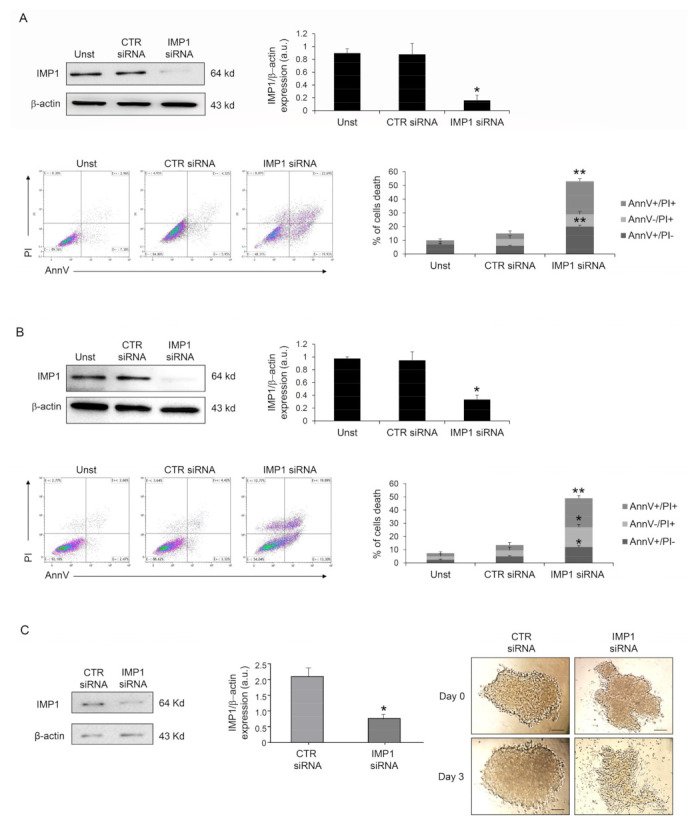
Down-regulation of IMP1 triggers cell death in PDAC cells and three-dimensional spheroids. (**A**) Western blot showing the protein expression of IMP1 in Panc-1 cells unstimulated (Unst) or transfected with control or IMP1 siRNA (final concentration 25 nM, upper left panel); flow cytometry analysis of Panc-1 cells treated as indicated above for 48 h and stained with Annexin V (AnnV) and propidium iodide (PI) (lower left panels). Lower right panel, proportion of AnnV and/or PI-positive Panc-1 cells (mean ± SEM; AnnV + PI+ unst and CTR siRNA-treated cells versus IMP1 siRNA-transfected cells, * *p* = 0.01; ** *p*≤ 0.001, *n* = 3). (**B**) Upper panels, Western blot (right panel) and quantification (left panel) showing IMP1 protein expression in Mia PaCa-2 cells unstimulated (Unst) or transfected with control or IMP1 siRNA (final concentration 25 nM, upper left panel). Lower left panels, flow cytometry analysis of Annexin V (AnnV) and propidium iodide (PI)-positive MIA PaCa-2 cells treated as indicated above; lower right panel, quantification of AnnV and/or PI-positive MIA PaCa-2 cells (mean ± SEM; AnnV + PI + unst and CTR siRNA-treated cells versus IMP1 siRNA-transfected cells, * *p* = 0.01; ** *p* ≤ 0.001, *n* = 3). (**C**) IMP1 protein expression, analyzed by Western blotting, in Panc-1 spheroids cells transfected with control or IMP1 siRNA (final concentration 25 nM); * *p* = 0.01, *n* = 3. Right panels, representative images of Panc-1 spheroids over a 4-day treatment period. Control or IMP1 siRNA was applied once a day 0 (D0) at a concentration of 25 nM. [Scale bars 100 μm]. For the original image of Western blot, see the Supplementary Material.

Because IMP1 operates as a post-transcriptional fine-tuner that controls the expression of some important mRNA targets necessary for the regulation of cell proliferation and growth in several solid tumors, we evaluated whether IMP1 had a role in PDAC proliferation/survival. IMP1 protein expression was abrogated in human PDAC Panc-1 cells with a specific siRNA (Figure 2A), and cell treatment with the IMP1 siRNA for 48 h substantially increased their susceptibility to spontaneous cell death (Figure 2A). Similar results were also obtained in MIA PaCa-2 human pancreatic cancer cells (Figure 2B). Furthermore, treatment with BTYNB, an inhibitor of IMP1 RNA binding [21], had a similar effect on Panc-1 cell death (Supplementary Figure S1). The effects of IMP1 siRNA-induced cell death and viability were further evaluated in Panc-1 three-dimensional spheroids. IMP1 siRNA-treated spheroids exhibited a significant reduction in IMP1 expression and rapidly decreased in size and lost their three-dimensional structure (Figure 2C).

**Figure 3:**
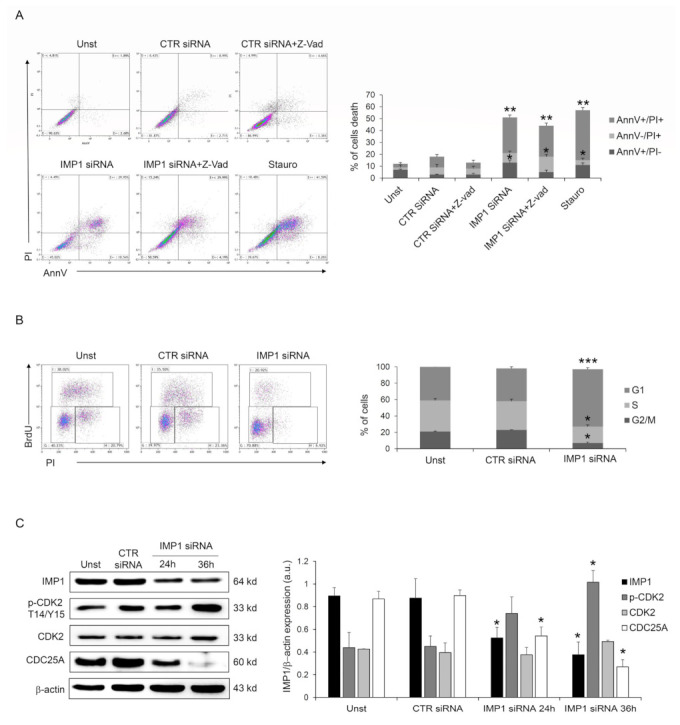
IMP1 abrogation induces cell-cycle arrest in Panc-1 cells. (**A**) Flow cytometry analysis of Annexin V (AnnV) and propidium iodide (PI)-positive Panc-1 cells pre-treated with pan-caspase inhibitor (Z-VAD) and treated or not with control or IMP1 siRNA for 60 h (mean ± SEM; AnnV + PI + unst and CTR siRNA-treated cells versus IMP1 siRNA-transfected cells, ** *p* ≤ 0.01; Z-VAD-cells and Z-VAD CTR siRNA-treated cells versus Z-VAD IMP1 siRNA-treated cells, * *p* ≤ 0.05, *n* = 4). (**B**) Representative flow cytometric analysis and cumulative data of cell-cycle progression in Panc-1 cells either left untreated (Unst) or incubated with control or IMP1 siRNA for 40 h (mean ± S.D. of 4 experiments, CTR siRNA-treated cells versus IMP1 siRNA-transfected cells *** *p* = 0.001, * *p* = 0.01). (**C**) Western blot (right) and quantitative analysis of IMP1, p-CDK2, CDK2, *CDC25A*, and b-actin expression in total protein extracts of Panc-1 cells left untreated (Unst) or transfected with control or IMP1 siRNA for 24 or 36 h of culture. Values are expressed in arbitrary units (a.u.) (mean ± S.E.M. of all experiments, CTR siRNA-treated cells versus IMP1 siRNA-transfected cells * *p* ≤ 0.05, *n* = 4). For the original image of Western blot, see the Supplementary Material.

Next, we investigated the IMP1 knockdown-dependent cell death mechanism, analyzing if it was a caspase-dependent death as a first step. However, pre-treatment with Panc-1 pan-caspase inhibitor (Z-VAD) did not change IMP1 siRNA-generated cell death (Figure 3A), suggesting that IMP1 affects PDAC cell survival without modifying the typical caspase-dependent cell death pathway. Moreover, the fraction of viable cells was unchanged by IMP1 silencing at 24/36 h (Supplementary Figure S2), indicating that IMP1 siRNA-mediated cell death might be secondary to cell growth arrest. IMP1 siRNA treatment in Panc-1 cells affected cell-cycle progression and triggered a gradual accumulation in the amount of cells in the G1 phase and a decrease in S and G2 phases (Figure 3B), indicating that DNA duplication is stopped following IMP1 silencing. The cyclin/cyclin-dependent kinase (CDK) complexes, formed at specific times, coordinate DNA duplication and mitosis in eucaryotic cells, and these events are required for cell-cycle progression and mitosis [22]. CDK2 activation is a crucial checkpoint for mitotic onset and progression from G1 to S phase. The biochemical function of CDK is regulated by both inhibitory and activating phosphorylation, and the CDK–cyclin complex is repressed by Thr-14 and Tyr-15 phosphorylation within the ATP-binding pocket [22]. Panc-1 silenced for IMP1 showed a marked phosphorylation of CDK2 on Thr-14 and Tyr-15 residues (Figure 3C). CDC25 family proteins, a specific group of phosphatases that dephosphorylate, are the master regulator of CDK–cyclin activation [23]. In detail, *CDC25A*, CDC25B, and CDC25C are three elements of the human CDC25 family, and *CDC25A* is involved in controlling the advancement through the S phase by controlling CDK-2 activation, while the transition from G2 to mitosis is controlled by CDC25B and CDC25C. Therefore, we analyzed whether silencing of IMP1 modulated *CDC25A* protein expression. As indicated in Figure 3C, IMP1 silencing is followed by a significant reduction in *CDC25A* expression in Panc-1 cell line (Figure 3C). Given our results on the depletion of IMP1 in the PDAC cell cycle, we analyzed the combinatorial effect of IMP1 inhibition with some chemotherapeutic agents used to treat PDAC patients that alter cell-cycle progression and DNA synthesis. Treatment of Panc-1 cells with IMP1 siRNA increased the sensibility of PDAC cells to 5-fluoruoracil and oxaliplatin, suggesting that IMP1 knockdown could potentially be deployed as an anticancer drug in combinatorial approaches with chemotherapeutic drugs (Supplementary Figure S3). Taken together, these findings suggest that IMP1 silencing was accompanied by a clear reduction in *CDC25A* expression, thus affecting cell-cycle progression and influencing PDAC cell proliferation and survival.

### 3.3. IMP1 Binds CDC25A mRNA

**Figure 4:**
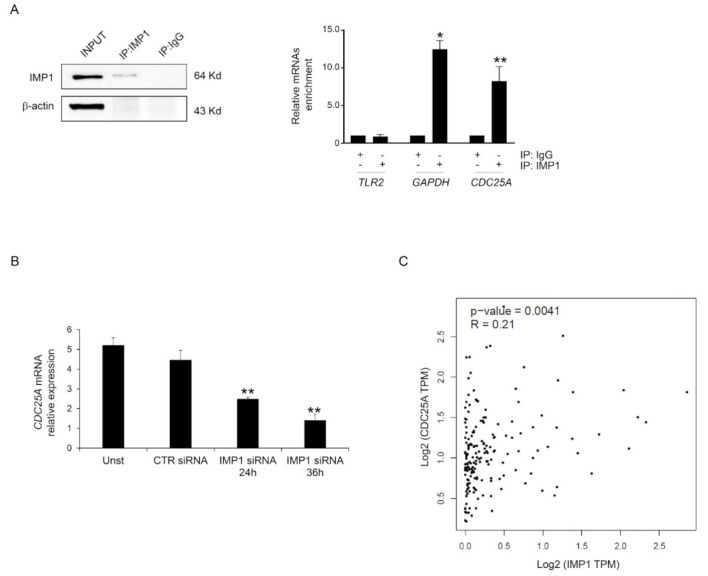
IMP1 binds *CDC25A* mRNA. (**A**) Left, Western blotting showing IMP1 immunoprecipitation from Panc-1 cells. Input (1:10) of total protein isolates, IMP1 immunoprecipitation (IP: IMP1), and mock immunoprecipitation (IP: IgG). Right inset, IMP1-mRNA enrichment in IMP1 immunoprecipitation/total protein isolated from Panc-1 cells was analyzed using real-time PCR. The *TLR2* and *GAPDH* mRNAs were used as negative and positive controls, respectively (mean ± SEM; IP: IMP1 versus IP: IgG * *p* ≤ 0.01, ** *p* ≤ 0.03; *n* = 3). (**B**) Using real-time PCR, *CDC25A* mRNA levels were analyzed in Panc-1 cells left untreated (Unst) or transfected with CTR or IMP1 siRNA for 24 or 36 h (Unst and CTR siRNA-treated cells versus IMP1 siRNA-transfected cells, ** *p* ≤ 0.03; *n* = 3). (**C**) Scatter blot of the correlation between IMP1 and *CDC25A* in human PDAC tissue using the GEPIA database. For the original image of Western blot, see the Supplementary Material.

Based on these data, we investigated the possibility that IMP1 binds the mRNAs encoding *CDC25A*. Primarily, we analyzed whether *CDC25A* protein inhibition mediated by IMP1 siRNA treatment resulted from variations in the expression of *CDC25A* mRNA. POSTAR3 is a database for investigating regulation at a post-transcriptional level based on high-throughput sequencing data from human tissue and provides the largest binding site group of binding sites of RPB and their potential mRNA targets [24]. We screened the POSTAR database, and *CDC25A* mRNAs were identified as potential targets of IMP1 (http://111.198.139.65/BindingSiteRecords.html?RBP=IGF2BP1&geneName=CDC25A&species=human&protocol=PAR-CLIP,PARalyzer, accessed on 1 October 2023). To further confirm this possible binding, IMP1 was immunoprecipitated from the total protein extracted from Panc-1, and the connection of *CDC25A* mRNAs was examined with real-time PCR (Figure 4A). Significant enrichment was discovered in the Panc-1 cell IMP1 complex for *CDC25A* and for *GAPDH* that was used as positive control but not for *TLR2* transcript, which was used as a negative control (Figure 4A). To assess whether IMP1 is crucial to maintaining reduced levels of *CDC25A* transcripts in PDAC, we explored *CDC25A* mRNA expression in a Panc-1 cell line treated with or without IMP1 siRNA. Efficient silencing of IMP1 in Panc-1 inhibited IMP1 expression and was correlated with *CDC25A* down-regulation at mRNA levels (Figure 4B). Using the available online tool, we explored the correlation between IMP1 and *CDC25A* expression. There was a positive interrelationship between IMP1 and *CDC25A* expression in human PDAC tissue (Figure 4C). These results indicate that IMP1 binds to the *CDC25A* mRNA and therefore positively influences *CDC25A* production.

### 3.4. IMP1 Knockdown Decreases In Vivo Growth of Panc-1-Derived Xenografts

**Figure 5:**
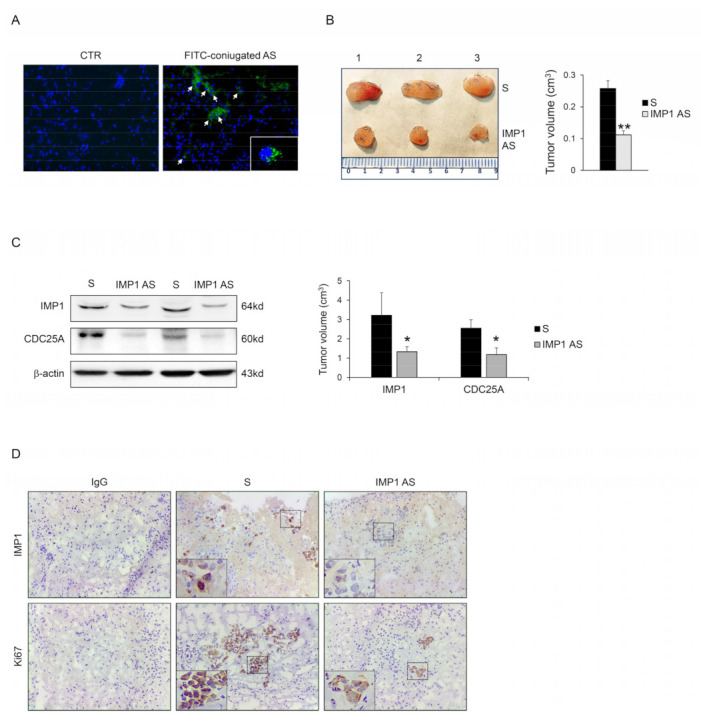
IMP1 controls the growth of Panc-1-derived tumors in vivo. (**A**) Fluorescein isothiocyanate (FITC)-conjugated IMP1 AS oligonucleotide in Panc-1-derived xenografts generated in Rag1−/− mice treated with one intraperitoneal injection of either control (PBS) or AS oligonucleotide (100 μg) (DAPI (blue) and FITC)-conjugated IMP1 AS (green)). (**B**) Representative images (left panel) and relative graph (right panel) displaying Panc-1-derived tumors volume taken from animals injected with control oligonucleotide (S) or IMP1 antisense (AS) oligonucleotide. Data are shown as mean ± S.D., which were comprised of 5 mice/group. (** *p* < 0.03; S-treated versus AS-treated mice, *n* = 3). (**C**) Representative blot (left panels) and relative graph (right panel) showing IMP1, *CDC25A*, and p-CDK2 protein expression in Panc-1-derived xenografts induced in Rag1−/− mice and treated as indicated above. β-actin was used as a loading control (* *p* < 0.01). (**D**) Representative images showing immunohistochemistry for IMP1 (upper panels) and proliferating Ki67-stained sections (lower panels) in tumors excised from the two different groups of mice. [Scale bars 100 μm]. For the original image of Western blot, see the Supplementary Material.

We examined the capacity of IMP1 to control cancer progression in vivo. Panc-1 cells were injected subcutaneously into RAG-1-deficient mice. After 7 days, mice were treated with an intraperitoneal dose of either phosphate-buffered saline (PBS; CTR) or fluorescein isothiocyanate (FITC)-conjugated IMP1-antisense oligonucleotide. After 24 h, mice were scarified, and the presence of FITC-IMP1 AS oligonucleotide with immunofluorescence was assessed in tumor tissue. FITC-IMP1 AS was taken up by cancer cells in the examined tumor tissue (Figure 5A), therefore, mice were treated daily by IP injection with either control sense (S) or IMP1 AS oligonucleotide (both at 100 mg/mouse) starting one week after the Panc-1 inoculation until sacrifice (day 21). No weight loss was detected in either treated group, and all mice remained alive until the end of the experiment. The Panc-1-originated cancer manifested in all animals, but mice that were treated with IMP1 AS oligonucleotide had a significantly reduced cancer volume (Figure 5B). Mice injected with IMP1 AS oligonucleotide but not with S had down-regulated IMP1 protein expression. Consistent with our in vitro results, silencing of IMP1 reduced *CDC25A* expression (Figure 5C,D). Finally, to examine whether the decrease in Panc-1-derived tumor volume detected in mice treated with IMP1 AS oligonucleotide was due to a cell proliferation reduction rate, we performed a Ki-67 cell proliferation assay (Ki67). Panc-1-derived tumors exhibited a reduced proportion of Ki67 positive cells, in agreement with the main findings of in vitro experiments.

## 4. Discussion

Convincing data reveal that cancer progression is supported by increased activation of several intracellular pathways that ultimately sustain the production/activation of proteins implicated in cell survival/cell death resistance and cell-cycle progression [4]. In the last decade, better knowledge of these altered intracellular signals has driven the development of therapeutic strategies. Here, using public databases, we have discovered a substantial increase in IMP1 expression in human PDAC tumors relative to the adjacent non-tumor area. In all stages observed using the UALCAN database, there is a high expression of IMP1 compared to the healthy area, suggesting that IMP1 expression increases from the initial stages of the disease even before lymph node metastasis, indicating that IMP1 could regulate one of the initial steps in the PDAC carcinogenesis process. Public database analyses revealed that up-regulation of IMP1 in PDAC was detected and correlated with a more aggressive clinic pathological phenotype and poorer prognoses, according to previous results demonstrating that IMP1 expression is a prognostic of poor survival [14]. More analysis are necessary to investigate the molecular mechanism that controls IMP1 expression in PDAC tissue; however, as reported in other human cancers, it is possible that, in addition to regulation at the level of transcription, IMP1 is tightly regulated at post-transcriptional levels by miRNAs such as let-7 or other RBPs such as LIN28, two molecules involved in the progression of PDAC [25,26,27,28].

We showed that IMP1 knockdown significantly reduced pancreatic cancer cell proliferation in human PDAC cell lines and three-dimensional spheroids and reduced tumor formation within in vivo Panc-1-derived xenografts. Silencing of IMP1 was related to cell-cycle block of PDAC cells in the G1 phase and consequent spontaneous cell death. IMP1-silenced PDAC cells showed a clear down-regulation of *CDC25A*, a phosphatase that leads to cell-cycle progression through CDK2 dephosphorylation and blocking of the activity of the dephosphorylation of CDK2-cyclin complex. Interestingly, inhibition of *CDC25A* by IMP1 siRNA appeared at the protein and mRNA levels, suggesting that IMP1 controls *CDC25A* at the transcriptional level. Specific immunoprecipitation analysis showed that IMP1 binds the mRNA of *CDC25A*, indicating that IMP1 plays a controller role in cell-cycle progression affecting *CDC25A* synthesis. Thus, for the first time, we detected in our study the binding between *CDC25A* mRNAs and the IMP1 complex, suggesting that IMP1 could regulate their metabolism. Using the POSTAR3 database to estimate mRNA targets, we found that *CDC25A* mRNA represents a potential IMP1 binding site. But this is an analytical method, and RBP could make for a complicated system to manipulate mRNA expression at the post-transcriptional point [29]; therefore, further studies should explore how IMP1 could control *CDC25A* metabolism or stability after mRNA binding. Our data are also supported by several recent papers that performed RNA-sequencing of several cancer cell lines after IMP1 knockdown, and *CDC25A* was found to be significantly down-regulated in IMP1-silenced cell lines including Panc-1 cells [30,31,32]. Furthermore, our results are consistent with previous articles demonstrating that down-regulation of IMP1 activity suppresses cell growth in neuroblastoma, melanoma, and ovarian cancer cells and has a synergistic effect on controlling neuroblastoma cell proliferation in combination with cyclin-dependent kinase (CDK) inhibitors [21,33]. We do not exclude that other molecules could participate in controlling cell-cycle progression and *CDC25A* expression in PDAC cells, and it is reasonable that an optimal outcome is given by modulating multiple factors that contribute to blocking cell-cycle progression. Moreover, our findings expand previous results, revealing the crucial role of IMP1 in supporting oncogenic gene expression by stabilizing mRNAs involved in cancer cell-cycle progression [30]. Furthermore, the demonstration that IMP1 is strongly expressed in PDAC cells and that IMP1 knockout blocks PDAC cell growth validates and expands on previous data on the role of IMP1 in negatively affecting the prognoses of PDAC patients [14]. Additionally, the combination of IMP1 silencing with some of the routinely used chemotherapeutics is promising, as it can provide better efficiency at lower doses, resulting in less severe side effects. Resistance to 5-fluorouracil or oxaliplatin, two important anticancer drugs, is a serious challenge in the treatment of pancreatic cancer; therefore, the study of new molecules that can overcome such drug resistance may allow for the development of new combination chemotherapy protocols to improve the results for cancer patients.

## 5. Conclusions

PDAC is one of the most common and fatal cancers with very restricted therapeutic options. Important medical advancement in diagnostic investigations, surgical procedures, and systemic therapies is certain to increase the survival of patients affected by pancreatic cancer. A greater understanding of the cellular and molecular biology of pancreatic cancer, including new molecules involved in cell survival, could lead to potential and innovative curative approaches. In conclusion, our data indicate that down-regulation of IMP1 drives PDAC cell-cycle arrest and represents a novel and attractive strategy for overcoming cell proliferation in PDAC and cancer drug resistance.

## Data Availability

The data presented in this study are available in this article.

## Supplementary Materials

The following supporting information can be downloaded at: https://www.mdpi.com/article/10.3390/cancers15204983/s1. Figure S1. Down-regulation of IMP1 activity increases cell death in PDAC cells. Flow cytometry analysis of Panc-1 cells treated treated with 10 μM BTYNB or with a DMSO vehicle control for 48 hours and stained with Annexin V (AnnV) and propidium iodide (PI). One representative of 2 separate experiments are shown. Figure S2. Upper panels, flow cytometry analysis of Annexin V (AnnV) and propidium iodide (PI)-positive Panc-1 cells treated unstimulated (Unst) or transfected with control siRNA (36h) or IMP1 siRNA (final concentration 25 nM) for 24 or 36 hours; lower panel, quantification of AnnV and/or PI-positive Panc-1 cells (mean ± SEM; n = 3). Figure S3. IMP1 knockdown enhances the toxicity of chemotherapeutic drugs. Representative dot-plots (upper panels) and quatification percentage (lower panel) showing the AnnexinV- and/or propridiun iodide (PI)-positive Panc-1 cells pre-incubated with control or IMP1 siRNA (both final concentration 25 nM) for 12 h and then stimulated or not with 5-fluorouracil (5-flu, 10 μM final concentration) or oxaliplatin (Oxa, 10 M final concentration) for further 36 h. One representative of 2 separate experiments are shown.
