# Supplementary material for: Insulin-like Growth Factor II mRNA-Binding Protein 1 Regulates Pancreatic Cancer Cell Growth through the Surveillance of CDC25A mRNA
Source: Cancers (Basel). 2023 Oct 13;15(20):4983. doi: 10.3390/cancers15204983 (PMC10605367; doi:10.3390/cancers15204983)
Supplement: Supplementary file 1 [file cancers-15-04983-s001.zip › cancers-2552362-supplementary.pdf]

Uncropped western blots

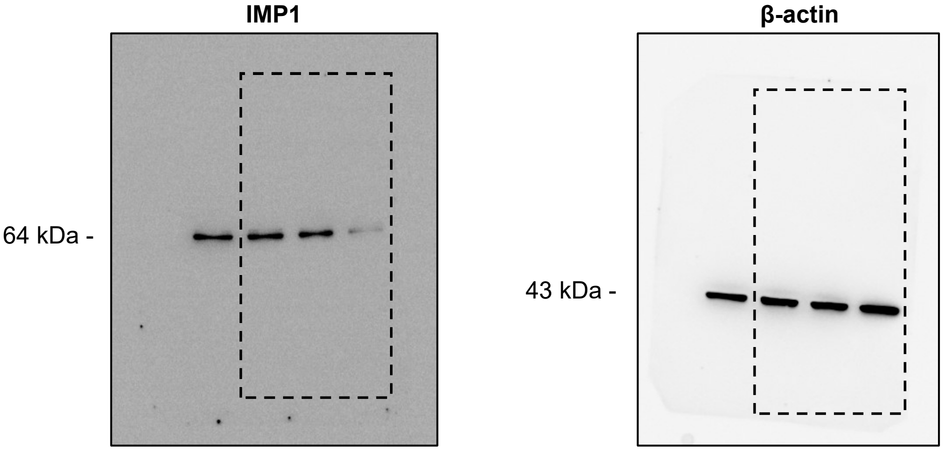

Figure 2A

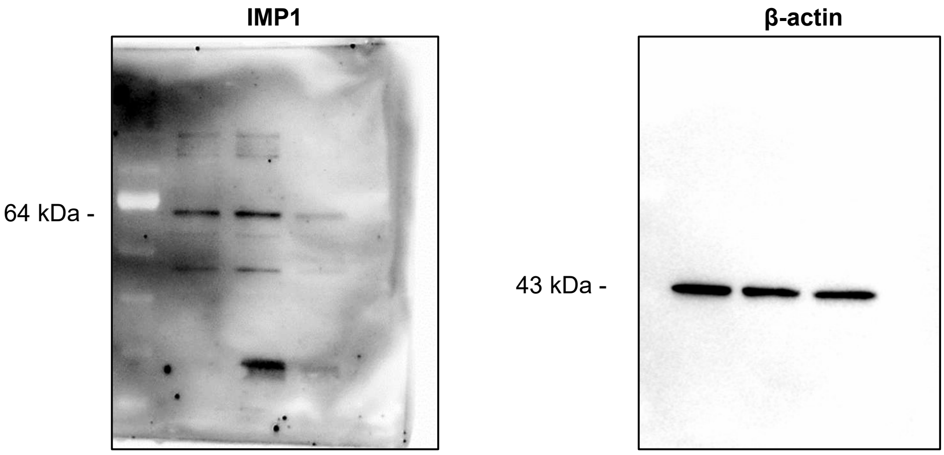

Figure 2B

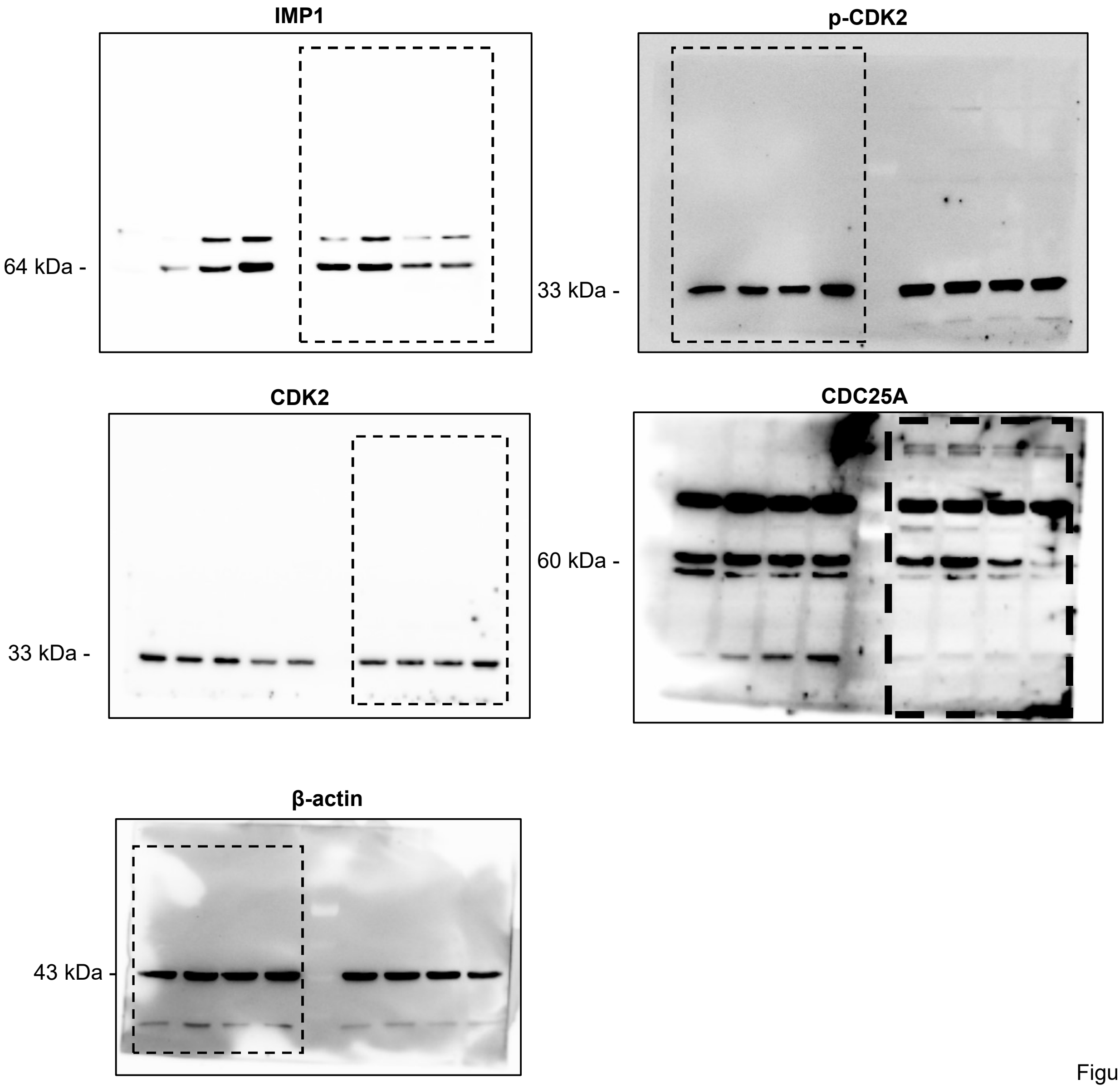
IMP1
64 kDa -
p-CDK2
33 kDa -
CDK2
33 kDa -
CDC25A
60 kDa -
β-actin
43 kDa -

Figure 3C

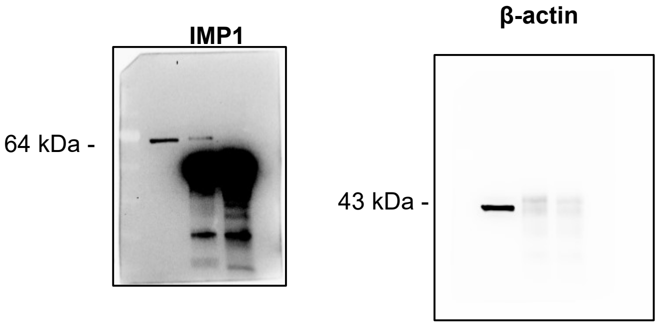

Figure 4A

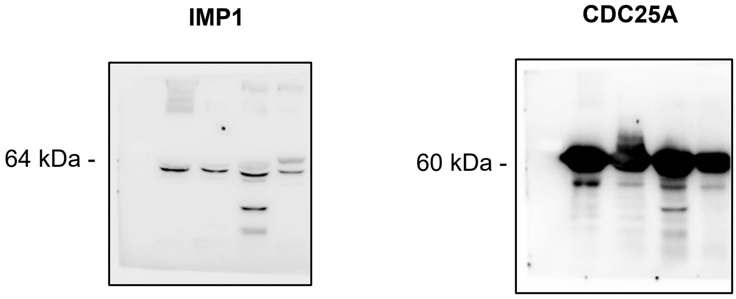

Figure 5C

Supplemental Materials

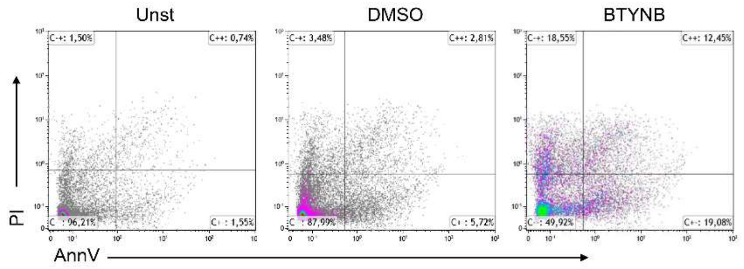

Supplemental Figure S1. Down-regulation of IMP1 activity increases cell death in PDAC cells. Flow cytometry analysis of Panc-1 cells treated treated with 10 uM BTYNB or with a DMSO vehicle control for 48 hours and stained with Annexin V (AnnV) and propidium iodide (PI). One representative of 2 separate experiments are shown.

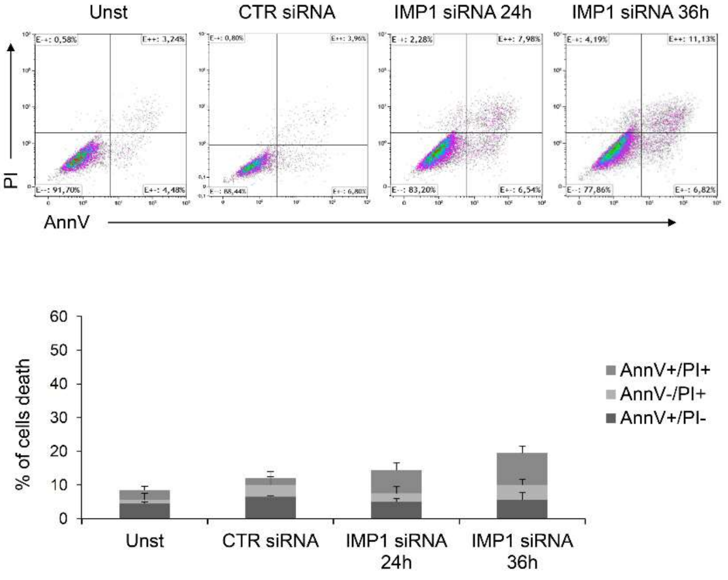

Supplemental Figure S2. Upper panels, flow cytometry analysis of Annexin V (AnnV) and propidium iodide (PI)-positive Panc-1 cells treated unstimulated (Unst) or transfected with control siRNA (36h) or IMP1 siRNA(final concentration 25nM) for 24 or 36 hours; lower panel, quantification of AnnV and/or PI-positive Panc-1 cells (mean ± SEM; n = 3).

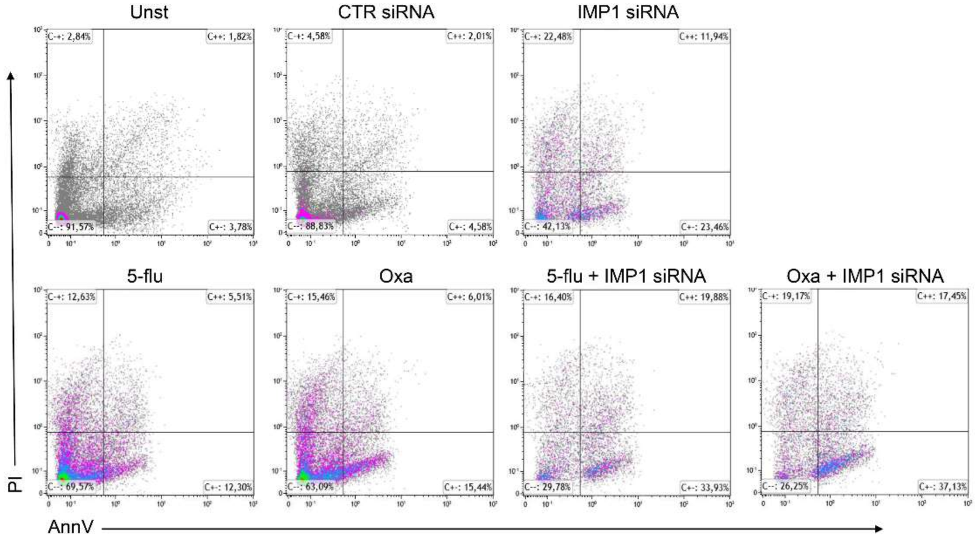

Supplemental Figure S3. IMP1 knockdown enhances the toxicity of chemotherapeutic drugs. Representative dot-plots (upper panels) and quatification percentage (lower panel) showing the AnnexinV- and/or propridiun iodide (PI)-positive Panc-1 cells pre-incubated with control or IMP1 siRNA (both final concentration 25 nM) for 12 h and then stimulated or not with 5-fluorouracil (5-flu, 10μM final concentration) or oxaliplatin (Oxa, 10μM final concentration) for further 36 h. One representative of 2 separate experiments are shown.
